# Vancomycin gene selection in the microbiome of urban *Rattus norvegicus* from hospital environment

**DOI:** 10.1093/emph/eow021

**Published:** 2016-07-12

**Authors:** Thomas Arn Hansen, Tejal Joshi, Anders Rhod Larsen, Paal Skytt Andersen, Klaus Harms, Sarah Mollerup, Eske Willerslev, Kurt Fuursted, Lars Peter Nielsen, Anders Johannes Hansen

**Affiliations:** 1 Centre for GeoGenetics, Natural History Museum of Denmark, University of Copenhagen, Copenhagen, DK-1350, Denmark;; 2 Center for Biological Sequence Analysis, Department of Systems Biology, Technical University of Denmark, Kemitorvet, 2800, Kgs, Lyngby, Denmark;; 3 Department of Microbiology & Infection Control, Statens Serum Institut;; 4 Department of Autoimmunology and Biomarkers, Statens Serum Institut, Copenhagen S, DK-2300, Denmark

**Keywords:** *vanb*, vancomycin, rats, antibiotics, *Rattus*, wild, metagenomics, selection

## Abstract

Background and objectives: Widespread use of antibiotics has resulted in selection pressure on genes that make bacteria non-responsive to antibiotics. These antibiotic-resistant bacteria are currently a major threat to global health. There are various possibilities for the transfer of antibiotic resistance genes. It has been argued that animal vectors such as *Rattus norvegicus* (*R. norvegicus*) living in hospital sewage systems are ideal for carrying pathogens responsible for fatal diseases in humans. Methodology: Using a metagenomic sequencing approach, we investigated faecal samples of *R. norvegicus* from three major cities for the presence of antibiotic resistance genes. Results: We show that despite the shared resistome within samples from the same geographic locations, samples from hospital area carry significantly abundant vancomycin resistance genes. Conclusions and implications: The observed pattern is consistent with a selection for vancomycin genes in the *R. norvegicus* microbiome, potentially driven by the outflow of antibiotics and antibiotic-resistant bacteria into the wastewater systems. Carriage of vancomycin resistance may suggest that *R. norvegicus* is acting as a reservoir for possible transmission to the human population.

## BACKGROUND AND OBJECTIVES

The clinical burden of infections caused by antibiotic-resistant pathogenic bacteria is an increasing challenge worldwide. Especially the nosocomial infections with multi-resistant bacteria are problematic because of the rising difficulties in targeted treatment that result in increased morbidity and mortality particularly in immunocompromised patients [1,2]. For four decades, the preferred choice of antibiotic drug to target methicillin-resistant *Staphylococcus aureus* (MRSA) has been vancomycin [3]; this has likely contributed to an increase in the number of vancomycin-resistant *Enterococcus* sp. (VRE) as well as vancomycin-intermediate-resistant *S. aureus* and vancomycin-resistant *S. aureus* strains identified in patients [4].

Resistance to vancomycin was first reported in *Enterococci* sp. in 1988 [5, 6] and is conferred by *van* operons, where *vanA* and *vanB* are clinically relevant and most investigated *van* operons [7]. Both *vanA* and *vanB* operons are mobilizable. Although *vanA* is typically located in Tn*1546*-like transposable elements that frequently reside on plasmids [8], *vanB* is often a part of a larger conjugable chromosomal element (Tn*1549*) [9, 10] but has also been observed on plasmids [11]. The pathogenesis of VRE ranges from infections of the urinary tract, biliary tract and wounds to severe bacteraemia and endocarditis, frequently linked to fatal outcome [12, 13]. VRE are more difficult to treat than the antibiotic susceptible species and therefore VRE infections are more often correlated with poorer prognosis and increased number of hospitalization days [14–17]. The resistance rate of clinical VRE in the period from 1995 to 2002 has increased from ˜47 to ˜70% in the USA [1], likely accounted by an increased use of vancomycin and other antibiotics in hospitals.

The use of antibiotics in the hospitals can be traced in the wastewater system of the hospitals, where both antibiotics and antibiotic-resistant bacteria are readily detected [18–22]. There is a risk that the antibiotic-resistant bacteria in the wastewater systems can interfere with the local ecosystem. First, antibiotics in the environment can select for resistant bacteria. Second, resistance traits can spread horizontally to the locally adapted sensitive bacteria when resistance functions are mobilizable. The gut of *Rattus norvegicus* (*R. norvegicus*) has been hypothesized to act as an incubator for antibiotic-resistant bacteria from the hospitals [23]. In this case, *R. norvegicus* could migrate and carry the antibiotic resistance genes or possibly carry mobile genetic elements acquired from the hospital wastewater systems.

In this study, we compare *R. norvegicus* faecal samples from hospital and non-hospital environments using a metagenomics DNA sequencing approach. We show that the faeces of *R. norvegicus* from hospital environment have elevated levels of vancomycin resistance genes.

## METHODOLOGY

Faecal samples were collected from urban areas of Malaysia, Hong Kong and Denmark. All Danish samples from wild rats (*n* = 20) were collected from four locations within the Copenhagen area: Egedal municipality (EM) (*n* = 3), Copenhagen University Hospital (CUH) (*n* = 6), Botanical Garden of Copenhagen (BGC) (*n* = 2) and Amager East (AE) (*n* = 9). In addition, five samples were collected in Kuala Lumpur (KLU), Malaysia, one in Kuala Langat, Malaysia and two samples were obtained from Hong Kong, China. Freshness of the samples was assessed by visual and tactile inspection. Rat fecal matter is easy to recognize visually. However, we performed a metabarcoding study on six of the samples, data published elsewhere [24], to confirm that they had *R. norvegicus* origins. The samples collected in Asia were shipped at ambient temperature in Falcon tubes and immediately frozen upon arrival. The samples from Denmark were frozen at –20°C within 24 h of collection.

The frozen faecal samples were vortexed vigorously in 800 µl of PBS for 1 min and incubated at room temperature for 30 min. Following the incubation, the samples were re-vortexed vigorously for a minute and then centrifuged at 12 000 g for 5 min. The supernatant was split into three aliquots of 160 µl and subsequently passed through 0.22 µm sterile filters at 6000 × g for 5 min. Each of the three filtrates were nuclease treated using 14 µl Turbo DNase (2 U/ul)(Ambion), 6 µl Baseline ZERO DNase (1 U/ul) (Epicentre), 6 µl RNase Cocktail (Ambion), 8.5 µl sterile water and 20.5 µl 10 × Turbo buffer in a total volume of 205 µl and incubated at 37°C for 2 h. The three aliquots were pooled and nucleic acid extracted using the QIAamp Viral RNA Mini Kit (Qiagen), followed by the addition of 1 µl RNase Out (Invitrogen) to the extract. Indexed DNA libraries were subsequently prepared using Nextera XT DNA Sample Preparation kit (Illumina), according to the manufacturers' guidelines. All subsequent sequencing was performed by 100 bp paired-end sequencing on an Illumina HiSeq 2000.

Raw reads from the HiSeq platform were demultiplexed using Novobarcode (http://www.novocraft.com, vBeta-0.8). For each sample, AdapterRemoval (v1.1) [25] was used to trim low quality bases, to remove adapter sequences from paired-end reads and to merge paired-end reads overlapping with more than 11 nucleotides.

The sequences were aligned to the ARG-ANNOT [26] nucleotide database using Bowtie2 [27] (–no-unal –end-to-end -q) and only perfect matches were used for further analyses. All matches were masked using Dustmasker (v1.0.0) [28] and read matches with less than 20% low-complexity nucleotide sequence and longer than 75 nucleotides were considered a real match and counted. Genes with less than ten reads mapping to them (*n* = 297) from all samples were excluded.

A global mean normalization was applied to the remaining 240 313 reads that mapped to the nucleotide sequences in the ARG-ANNOT database [26]. Empirical Bayes moderated t-tests were obtained using limma [29]. *P*-values were adjusted using the Bonferroni method of multiple-testing correction. To further characterize genes differentially distributed between hospital and non-hospital samples, we performed two-dimensional hierarchical clustering using Euclidean distance measure.

To identify genes shared among sample locations, a gene was considered present in a location if at least one read mapped to the gene sequence. Assembly of the reads was performed using Ray Meta (v2.2.0 default settings) [30] and contigs were mapped to the ARG-ANNOT database using bowtie2 and the contigs with a match were aligned using BLASTn (v2.2.29+ default settings) to verify their origin. To establish the microbial composition of the samples we applied MetaPhlAn (v1.7.7 default settings) [31] on the reads of the samples.

## RESULTS

**Figure 1.**
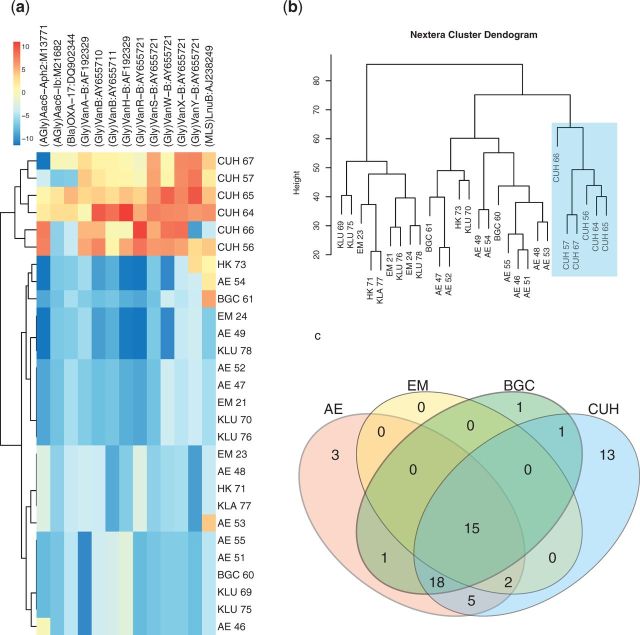
Summary of resistome comparison. **(a)** Two-dimensional hierarchical clustering of significantly differentially abundant genes between hospital and non-hospital environments. Hospital samples (labelled CUH) show higher levels of vancomycin resistance genes compared to the non-hospital samples. Abbreviations are EM, Denmark, CUH, Denmark, BGC, Denmark, AE, Denmark, KLU, Malaysia, Kuala Langat, Malaysia (KLA) and Hong Kong, (HK). **(b)** Hierarchical clustering of normalized read counts of the ARG-ANNOT mapped genes, using Euclidean distance method. **(c)** A Venn diagram showing the number of genes shared among sample locations in Copenhagen area

Rat faecal samples were collected in the vicinity of Copenhagen University Hospital (CUH), and several urban, non-hospital locations in Copenhagen, Malaysia and Hong Kong. DNA from the samples was isolated and sequenced using a metagenomic approach and a total 240 313 reads were mapped onto the ARG-ANNOT [26] database that comprises 1689 curated antibiotic resistance genes of which 89 are vancomycin resistance genes. Read mapping to the antibiotic resistance genes showed that 63 of these 1689 genes had at least 10 mapped reads across samples, and they were considered hits (Supplementary Fig. S1). There were 2697 unique reads mapping to vancomycin resistance genes. A student’s *t*-test demonstrated a significant difference between the samples from the hospital area and all other locations in terms of the total uniquely mapped reads. Out of the 63 genes with at least 10 mapped reads, 13 genes had significantly higher amounts of mapped reads in hospital compared to non-hospital samples. Nine of these significantly abundant genes were *vanB* genes. Two-dimensional hierarchical clustering of these genes with all samples demonstrated high levels of vancomycin genes in the CUH samples (Fig. 1a). Hierarchical clustering of all normalized reads mapping to ARG-ANNOT genes showed a clear clustering among CUH samples (Fig. 1b). To establish the common resistome and shared antibiotic resistance genes, we grouped the samples based on locations and compared the read counts of antibiotic resistance genes. If a gene had at least one read match, it was considered to be present. A majority of genes (15) were shared among all locations in the Copenhagen area (Fig. 1c), whereas 13 genes were specifically present in CUH samples. The urban Copenhagen area (AE, CUH, BCG) had a shared resistome (18 genes) that was not shared with the rural EM samples. In summary, there clearly appeared to be a shared resistome among samples in the Copenhagen area, but the samples from CUH carried a specific set of genes (13), that were not present in other samples from Copenhagen (Fig. 1c).

To obtain more information on the underlying genomic sequences represented by all reads, we *de novo* assembled the reads into contigs. The contigs resembling vancomycin-resistance genes were short (100–750 nucleotides), but the majority of them mapped with high similarity to the *vanB* operon SAU16 (Acc:KF823968) or SAU28 (Acc:KF823969), which is typically located on the transposon Tn*1549.* We therefore mapped all reads from the hospital samples to the Tn*1549* transposon (accession number: NG_035288) to see the combined read distribution (Supplementary Fig. S2). The consensus sequence of the mapped reads had 99% identity to Tn*1549*, an average read depth of 54 and the entire Tn*1549* sequence had a read depth of at least one. Furthermore, when mapping to MetaPhlAn marker genes in samples across the geographical locations, we observed low quantities of *Enterococcus faecalis* and *Enterococcus faecium* (<0.5% in samples from AE, EM, HK, KLU and CUH) and *S. aureus* (< 0.08% in samples from AE, EM, BGC, KLU and CUH).

## CONCLUSIONS AND IMPLICATIONS

Recent investigations of antibiotic resistance genes in environmental, human gut and faecal samples from wildlife have revealed the presence of a variety of antibiotic resistance genes, collectively called a resistome [32–34]. The resistome from urban *R. norvegicus* faecal samples from seven locations in three major cities around the world revealed the presence of a variety of genes associated with antibiotic resistance (Supplementary Fig. S1). The resistomes from the hospital locations clearly cluster together, whereas the other samples show no tendency of geographical clustering (Fig. 1a and b). A *t*-test analysis showed significant differences between the resistome of hospital and non-hospital samples. Highly abundant vancomycin resistance genes present in the hospital samples primarily accounted for these differences (Fig. 1a and c).

Contigs mapping to the vancomycin resistance genes of the *vanB* genotype (Fig. 1a) had high similarity to the SAU28 operon and the nearly identical SAU16 operon in clinical *E. faecium* strains. One of the most common means of the transfer of antibiotic resistance genes between bacterial populations is horizontal gene transfer (HGT) via mobile genetic elements. The conjugative transposon Tn*1549* carries the *vanB* operon as well as elements for HGT of the transposon by conjugation [10], allowing itself to spread to bacterial populations independent of other mobile elements. We found that a consensus sequence derived from all reads from the hospital-associated samples mapped to the complete transposon Tn*1549* with 99% identity. Although normally found in the chromosome, Tn*1549* has also been found on plasmids in clinical isolates of *E. faecalis* [11, 35], suggesting multiple ways of transfer of the *vanB* resistance traits.

Rodents' intestines may constitute an important reservoir of microbes including human pathogens such as MRSA and VRE [36, 37] and antibiotics present in their guts may favour colonization of intestines by opportunistic, antibiotic-resistant pathogens [38]. Additionally, exposure to low levels of antibiotics has been shown to drive the selection for antibiotic resistance genes in bacterial communities [39]. Hence, long-term exposure of rats to antibiotics could drive a positive selection for resistance genes in their guts. The hierarchical clustering of all normalized reads showed a clear clustering among CUH samples, indicating that *vanB* genes have been selected for in the guts of *R. norvegicus* near hospitals (Fig. 1a and b). Interestingly, high levels of vancomycin have been detected from the CUH wastewater [22] (median vancomycin concentration of 9.1 μg/l), which could be the main driver of the selection for *vanB* genes. The detection of *vanB* in hospital samples suggests that antibiotics used in hospitals and their subsequent spread to the environment through sewage might select for VREs in the rats' intestines.

As mentioned earlier, *Rattus* spp. are well-known carriers of bacterial pathogens like MRSA, *Leptospira*, *Streptobacillus moniliformis* etc [40, 41], and our results indicate presence of *Enterococcus* spp. as well. In addition, individual rats residing close to hospital environments have been shown to carry hundreds of plasmids in their gut microbiome [23]. It could therefore be of interest to explore if *R. norvegicus* can act as a vector of plasmids and transposons carrying vancomycin resistance genes to humans around hospitals.

**Figure 2.**
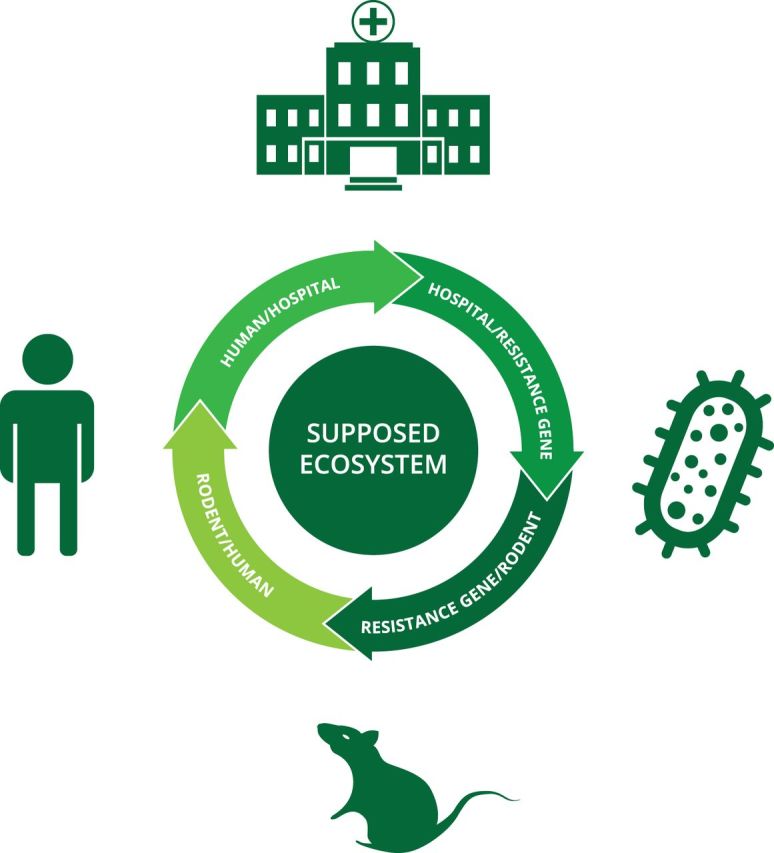
Proposed ecosystem. A proposed system of antibiotic resistance from hospitals acquired by bacteria that are carried further by rodents and passed on to humans that return to the hospital for treatment

Recently, risks associated with antibiotic resistance in the environment were ranked, and highest risk was attributed to antibiotic resistance genes on mobile elements that can be acquired or hosted by known human pathogens [42]. In this study, we highlight this risk by demonstrating significantly higher abundance of resistance genes exclusively in hospital samples and showing that these abundant genes are associated with mobile genetic elements. Furthermore, the risk situation can be assessed with an ecological perspective in which the ecological connectivity is deemed to be a major determinant of the horizontal transfer of antibiotic resistance [43]. Essentially, a donor and a recipient need to be in close contact, and still a subsequent transfer event between habitats would be low unless the recipient is under positive selection, which is the case in the presence of antibiotics [42]. We have discovered a high-risk situation that might be driven by the elevated number of antibiotic resistance genes and influx of antibiotics, as seen in the sewage from the hospital use, in combination with an environmental connectivity between wastewater, rats and humans. If humans are infected with vancomycin-resistant pathogenic bacteria spread by *R. norvegicus* as carrier, there could be a complete environmental loop from rodents to humans requiring hospitalization, and thus spreading the resistance genes further to other rodents or patients (Fig. 2).

A transmission route has been proposed for the spread of VRE between animals and humans, but it does not include the spread from sewage to wildlife and subsequently to humans [44]. In this study, we demonstrate that the transfer of antibiotic-resistant bacteria from hospital environment to rodents is a strong possibility. Several studies demonstrate antibiotics and antibiotic-resistant bacteria in sewage around hospital environments [18–22, 45]. One study showed a higher prevalence of extended-spectrum beta-lactamase-producing *Escherichia coli* in rats around hospital wastewater [46]. If elevated levels of antibiotics in the hospital wastewater are driving the selection of antibiotic resistance in an important vector like *R. norvegicus*, it is imperative to investigate the full extent of this phenomenon on a global scale. Besides hospitals, where the same antibiotic selection-driven ecosystem probably resides, farms with livestock also have an extensive use of antibiotics. Assessment of the spread of antibiotic resistance genes into important vector species in these areas will also be highly relevant for risk assessment. It would also be of outmost importance to determine what relevant pathways exist for spread of antibiotic resistance genes from *R. norvegicus* to the human microbiome, so that appropriate timely measures can be taken to limit the spread of fatal infections to humans. 

## Supplementary data

Supplementary data is available at *EMPH* online. 

## Supplementary Material

Supplementary Data
