# Supplementary material for: Vancomycin gene selection in the microbiome of urban Rattus norvegicus from hospital environment
Source: Evol Med Public Health. 2016 Jul 12;2016(1):219–26. doi: 10.1093/emph/eow021 (PMC4972940; doi:10.1093/emph/eow021)
Supplement: Supplementary Data [file eow021_Supp.zip › eow021-suppl_data/Supplementary_Fig_1.pdf]

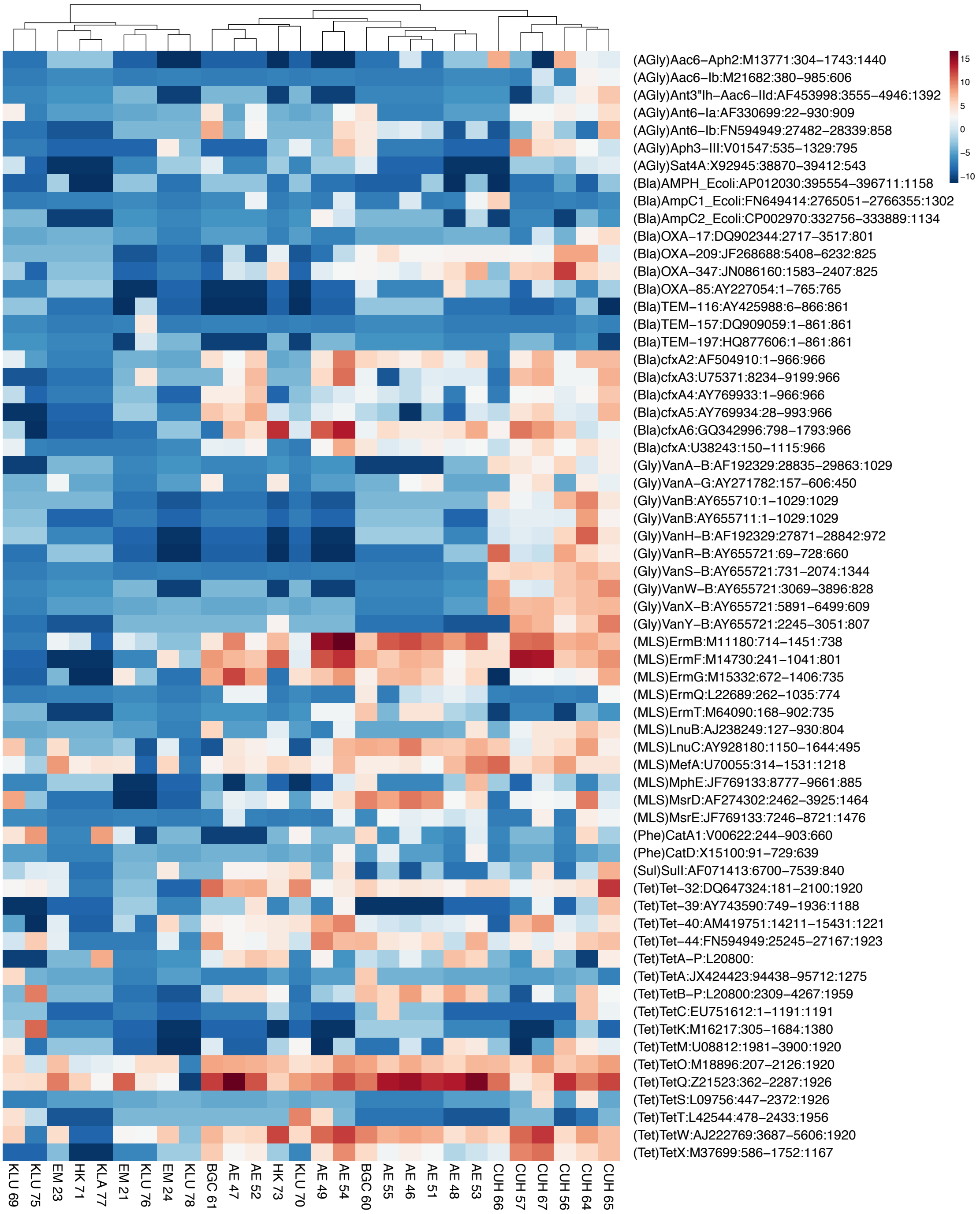

Supplementary Fig. 1: **The *R. norvegicus* resistome**. Two-dimensional hierarchical clustering of reads mapping to ARG-ANNOT genes. Hospital samples (CUH) show higher levels of vancomycin genes compared to the remaining non-hospital samples. Abbreviations are Egedal municipality (EM), Copenhagen University Hospital (CUH), Botanical Garden of Copenhagen (BGC), Amager East (AE), Kuala Lumpur (KLU), Kuala Langat (KLA) and Hong Kong (HK).
