# Supplementary material for: Vancomycin gene selection in the microbiome of urban Rattus norvegicus from hospital environment
Source: Evol Med Public Health. 2016 Jul 12;2016(1):219–26. doi: 10.1093/emph/eow021 (PMC4972940; doi:10.1093/emph/eow021)
Supplement: Supplementary Data [file eow021_Supp.zip › eow021-suppl_data/Supplementary_Fig_2.pdf]

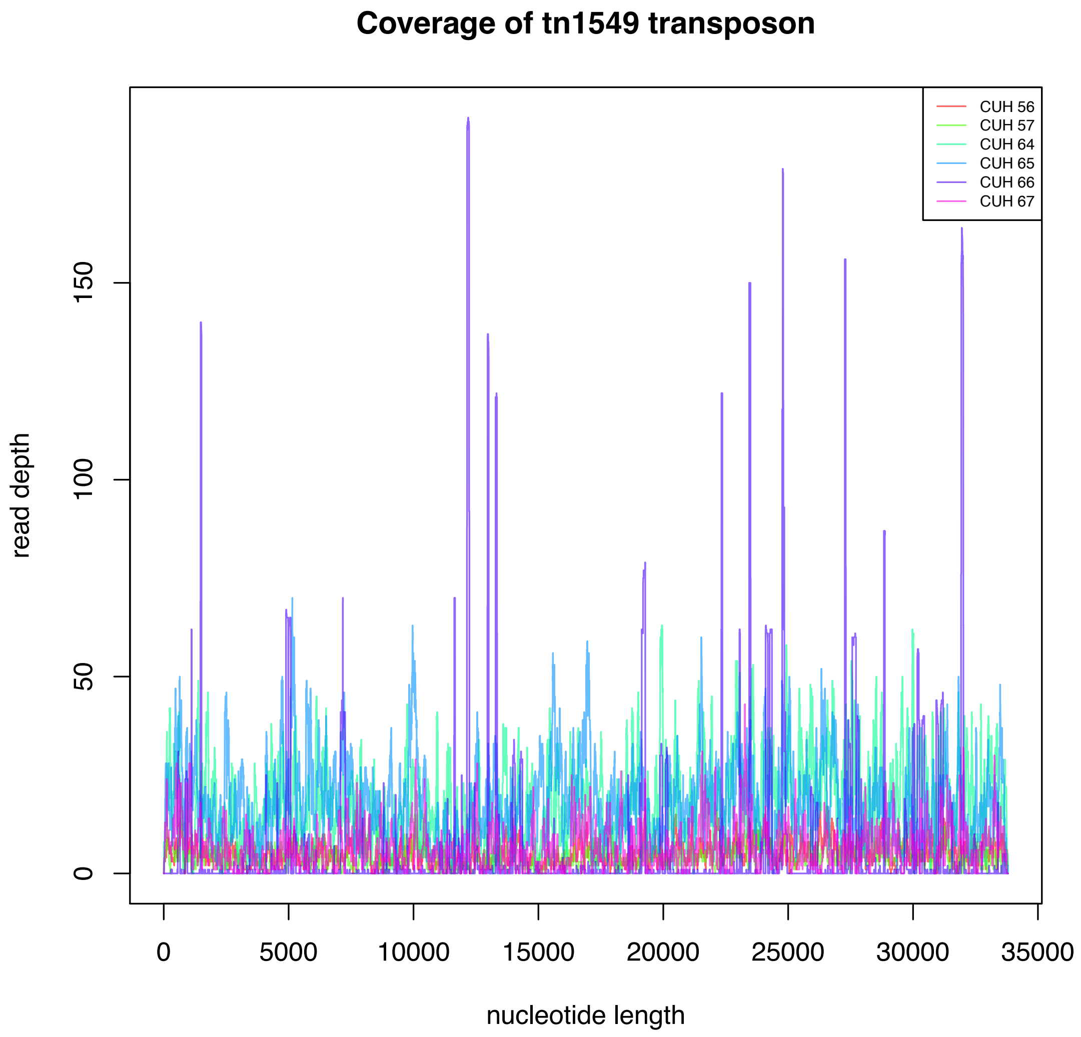

Supplementary Fig. 2: **Read coverage of transposon Tn*1549*.** Read coverage of transposon Tn*1549* in six rodent faecal samples collected from hospital environment.
